# Cucumber‐Derived Exosome‐Like Vesicles With Melanin‐Inhibitory Activity and an Acceptable in Vitro Safety Profile

**DOI:** 10.1111/jocd.70980

**Published:** 2026-06-11

**Authors:** Che‐Yuan Hsu, Wei‐Chen Chen, Horng‐Shen Chen, Yu‐Chen Liao, Cheng‐Han Hsieh, Donald Liu, Ya‐Wen Cheng

**Affiliations:** ^1^ Department of Dermatology National Taiwan University Hospital Taipei Taiwan; ^2^ Department of Research & Development Stem Biotechnology Inc. Taipei Taiwan; ^3^ Department of Dermatology Taipei Medical University, Shuang ho Hospital New Taipei Taiwan

**Keywords:** cucumber‐derived exosomes, melanin inhibition, plant‐derived nanovesicles, skin depigmentation

## Abstract

**Background:**

Skin hyperpigmentation disorders affect millions worldwide, driving demand for depigmentation products. However, many conventional skin‐lightening products contain dangerous ingredients, including mercury and unregulated hydroquinone.

**Aims:**

Plant‐derived exosome‐like vesicles represent promising natural therapeutic agents due to their biocompatibility and ability to deliver bioactive compounds. This study aimed to isolate and characterize cucumber‐derived exosome‐like vesicles (cEVs) and evaluate their melanin‐inhibitory effects and biosafety profile.

**Methods:**

Cucumber‐derived exosomes were isolated using differential centrifugation, filtration, and ultrafiltration. Physicochemical characterization was performed using nanoparticle tracking analysis (NTA), transmission electron microscopy (TEM), scanning electron microscopy (SEM), and SDS‐PAGE. The vesicular identity of purified cEVs was further validated by immunoblotting for established plant EV marker proteins PEN1 and TET8. Cytotoxicity and melanin‐inhibitory effects were assessed in MNT‐1 human melanoma cells. Melanogenic gene and protein expression of tyrosinase (TYR) and tyrosinase‐related protein 1 (TRP‐1) were assessed by qRT‐PCR and immunoblotting, respectively.

**Results:**

Purified cEVs exhibited physicochemical and morphological characteristics consistent with plant‐derived extracellular vesicles, including a mean diameter of 106.7 ± 1.4 nm, spherical morphology, intact membrane structures, and detectable expression of plant EV marker proteins PEN1 and TET8. The cEVs demonstrated no detectable cytotoxicity within the tested concentration range across concentrations of 3.9 × 10^8^ to 2.5 × 10^10^ particles/mL (> 95% MNT‐1 cell viability; 99.9% reconstructed human epidermis viability; classified as non‐irritating per UN GHS criteria). At the highest concentration, cEVs reduced melanin to approximately 75% of control, comparable to kojic acid and α‐arbutin, with immunoblotting confirming dose‐dependent downregulation of TYR protein expression consistent with the observed melanin‐inhibitory activity.

**Conclusions:**

Cucumber‐derived exosome‐like vesicles demonstrate concentration‐dependent melanin‐inhibitory activity without detectable cytotoxicity or skin irritation within the tested conditions, warranting further investigation as candidate naturally‐derived alternatives to conventional skin‐lightening products, warranting further preclinical investigation.

## 1 Introduction

Skin hyperpigmentation disorders, characterized by excessive melanin accumulation, represent a significant dermatological and cosmetic concern affecting millions of individuals worldwide [1, 2]. These conditions, including melasma, age spots, post‐inflammatory hyperpigmentation, and uneven skin tone, can substantially impact quality of life and psychological health [1]. The global skin lightening market has grown remarkably, from $8.8 billion USD in 2022 to a projected $15.7 billion by 2030, reflecting the increasing demand for depigmentation solutions [3]. However, many conventional skin‐lightening agents are associated with adverse effects and safety concerns [3, 4]. Numerous products contain dangerous and unregulated ingredients, including toxic levels of mercury and unmonitored concentrations of hydroquinone [3, 4]. These safety concerns highlight the critical need to develop novel, safer alternatives from natural sources with well‐established safety profiles.

Melanogenesis, the multistep enzymatic process of melanin biosynthesis, is primarily regulated through sequential catalytic reactions [5]. Tyrosinase (TYR), the rate‐limiting copper‐containing enzyme in melanogenesis, catalyzes two key reactions: the hydroxylation of L‐tyrosine to L‐3,4‐dihydroxyphenylalanine (L‐DOPA) and the subsequent oxidation of L‐DOPA to dopaquinone [6]. Tyrosinase‐related protein 1 (TRP‐1), also known as 5,6‐dihydroxyindole‐2‐carboxylic acid (DHICA) oxidase, plays a critical role in stabilizing tyrosinase and catalyzing the oxidation of DHICA to produce eumelanin, the brown‐black pigment responsible for skin coloration [5, 6, 7]. Given the central roles of TYR and TRP‐1 in melanogenesis, these enzymes have emerged as primary therapeutic targets for the development of depigmenting agents [8]. Currently available skin‐lightening agents include kojic acid, a fungal metabolite that inhibits tyrosinase activity by chelating copper ions at the enzyme's active site, and α‐arbutin (4‐hydroxyphenyl‐β‐D‐glucopyranoside), a glycosylated hydroquinone derivative that competitively inhibits tyrosinase [8]. Although clinically effective, concerns regarding their stability, cytotoxicity at high concentrations, and variable efficacy have motivated the search for safer, naturally‐derived alternative depigmentation approaches [8].

Recent extracellular vesicle research has revealed that plant‐derived exosome‐like vesicles represent a novel class of bioactive nanocarriers with therapeutic potential, particularly for cosmetic and dermatological applications [9, 10]. These lipid bilayer‐enclosed nanoparticles (30–200 nm) are naturally secreted by plant cells and can be isolated from commonly consumed edible plants and their juices [9, 10]. These vesicles encapsulate diverse bioactive cargoes including proteins, lipids, nucleic acids, and secondary metabolites that are transferred to recipient cells, thereby modulating various biological processes [9, 10]. Plant‐derived exosome‐like vesicles possess several properties relevant to therapeutic delivery, including gastrointestinal stability, a capacity for cellular uptake demonstrated in vitro, and documented low cytotoxicity in mammalian cell systems [11]. Plant‐derived vesicles exhibit diverse biological activities, including anti‐inflammatory, antioxidant, immunomodulatory, and anticancer properties, establishing their potential as multifunctional natural therapeutic agents for various applications including cosmetics [9, 10, 11].

Nanoparticles are increasingly utilized in cosmetic and dermatologic products due to their enhanced cellular uptake properties. Plant‐derived exosome‐like vesicles represent an innovative approach for hyperpigmentation treatment, uniquely combining nanoparticle advantages with natural product safety [12, 13]. These plant‐derived vesicles offer potential advantages over synthetic alternatives: low observed cytotoxicity in preclinical studies, low immunogenicity through regular dietary exposure, and established safety profiles from edible plants with long consumption histories [12, 13]. These attributes directly address the critical safety concerns associated with mercury‐containing products and unregulated synthetic chemicals in the current skin‐lightening market. Their lipid bilayer structures provide additional advantages, including efficient cellular uptake and effective intracellular delivery of multiple bioactive compounds for potential synergistic depigmentation effects. Furthermore, these vesicles can be produced cost‐effectively at scale from readily available plant sources.

Among plant sources, cucumber ( *Cucumis sativus* ) has been traditionally valued in skincare applications for its diverse beneficial properties [14, 15]. Its high water content provides instant hydration and strengthens the skin's moisture barrier while delivering cooling sensations that soothe sunburns and irritation [14]. Rich in minerals such as silica, potassium, and magnesium, cucumber exhibits cooling and potential anti‐aging effects [14]. Most notably, bioactive compounds can inhibit melanin synthesis, suggesting depigmentation potential; however, cucumber‐derived exosome‐like vesicles (cEVs) remain largely unexplored for such applications [14, 15].

In this study, we established a systematic protocol for the isolation and purification of cEVs and characterized their physicochemical properties. We investigated the biosafety and melanin‐inhibitory potential of purified cEVs using MNT‐1 human melanoma cells and examined their effects on key melanogenic genes TYR and TRP‐1. This work aims to provide preliminary in vitro evidence supporting their further evaluation as naturally‐derived depigmentation candidates.

## 2 Materials and Methods

### 2.1 Preparation of Cucumber‐Derived Exosome‐Like Vesicles

Cucumbers were purchased from a local supermarket in Taipei, Taiwan (supplier origin: Yunlin, Taiwan). Three randomly selected cucumbers (total weight: 339 g) were washed with tap water and then rinsed with deionized, distilled water (ddH_2_O). The washed cucumbers were peeled using a peeler (peeled weight: 270 g), rinsed again with ddH_2_O, cut into smaller pieces, and juiced using a commercial juicer (Model EX‐301SS, Kingpro, New Taipei, Taiwan). The raw juice was first centrifuged at 2600 × g for 20 min at room temperature. The supernatant was collected and subjected to a second centrifugation at 10000 × g for 20 min at 4°C. The resulting clear supernatant (180 mL) was filtered through 0.45‐μm polyethersulfone (PES) membranes (final volume: 178 mL). Subsequently, cEVs were purified using ultrafiltration. Briefly, the filtered cucumber juice was passed through a 100‐kDa molecular weight cut‐off (MWCO) PES membrane. After complete filtration, cEVs retained on the membrane were recovered by rinsing the membrane with sterilized 1× phosphate‐buffered saline (PBS) 50 times. The recovered suspension was centrifuged at 10000 × g for 10 min at 4°C to remove insoluble particles and debris. The cEVs‐containing supernatant was collected and stored at −80°C for downstream analyses.

### 2.2 Cell Culture

The highly pigmented human melanoma cell line MNT‐1 was purchased from the American Type Culture Collection (CRL‐3450, ATCC). Cells were cultured in Dulbecco's Modified Eagle's Medium (DMEM) supplemented with 20% fetal bovine serum (FBS), 10% AIM‐V medium, 0.1 mM non‐essential amino acids (NEAA; Gibco, Thermo Fisher Scientific), 100 units/mL penicillin, and 100 μg/mL streptomycin at 37°C in a humidified atmosphere containing 5% CO_2_. Upon reaching 90%–95% confluence, MNT‐1 cells were detached using 0.05% trypsin–EDTA (Gibco, Thermo Fisher Scientific) and passaged into cell culture flasks or multi‐well plates for subsequent experiments.

### 2.3 Nanoparticle Tracking Analysis (NTA)

The particle size distribution and concentration of cEVs were analyzed via NTA using a NanoSight NS300 instrument (Malvern Panalytical, Malvern, United Kingdom). Briefly, to achieve optimal tracking results, purified cEVs were diluted 1:1000 in sterile water to maintain 50–100 particles per frame. Measurements were performed under the following settings: camera type, sCMOS; camera level, 15; laser type, Blue488; slider shutter, 1206; slider gain, 366; viscosity, water. Three measurements were recorded as videos and analyzed using the built‐in NanoSight NTA software (version 3.4.003). Each sample was measured and analyzed in triplicate.

### 2.4 Transmission Electron Microscopy (TEM)

Purified cEVs were deposited onto a 300‐mesh copper grid with carbon‐coated Formvar film (cat. no. FCF300‐CU, Electron Microscopy Sciences, Hatfield, PA, USA) and incubated for 10–30 s. Excess sample was gently removed using blotting paper. The grid was negatively stained with 2% phosphotungstic acid (PTA), and excess liquid was removed using blotting paper. After staining, the grid was examined using a Hitachi H‐7100 transmission electron microscope (Hitachi High‐Technologies Corporation, Tokyo, Japan) operated at an accelerating voltage of 120 kV.

### 2.5 Scanning Electron Microscopy (SEM)

Purified cEVs were fixed with 2% paraformaldehyde and incubated at room temperature for 1 h. A 0.5‐μL aliquot of the fixed sample was deposited onto a plasma‐cleaned silicon wafer, allowed to air‐dry, and subsequently coated with a thin gold layer via sputtering. The prepared samples were imaged using a scanning electron microscope operated at an acceleration voltage of 10 kV with bias voltage applied. Image integration was performed to enhance the signal‐to‐noise ratio.

### 2.6 SDS‐Page

The protein concentration of purified cEVs was quantified using a bicinchoninic acid (BCA) protein assay kit (cat. no. JB04‐D001, T‐Pro Biotechnology, New Taipei City, Taiwan) according to the manufacturer's instructions. For SDS‐PAGE analysis, gels were prepared using the TGX Stain‐Free FastCast Acrylamide Kit (Bio‐Rad Laboratories Inc., Hercules, CA, USA). Purified cEVs (equivalent to 20 μg of total protein) were loaded and resolved on a 10% acrylamide gel under reducing conditions. After electrophoretic separation, proteins were stained with colloidal Coomassie blue.

### 2.7 Cytotoxicity Assay

To determine the potential cytotoxicity of purified cEVs, MNT‐1 cells were seeded in 96‐well cell culture plates at a density of 3000 cells/well in 50 μL of culture medium. After cell attachment, the cells were treated with 50 μL of medium‐diluted cEVs for 72 h. Following treatment, 20 μL/well of CellTiter 96 AQueous One Solution Reagent (Promega Corporation, Madison, WI, USA) was added, and cells were incubated at 37°C for 2 h in a humidified atmosphere containing 5% CO_2_. Cell viability was determined by measuring the absorbance of the MTS formazan product at 490 nm using a microplate reader.

### 2.8 In Vitro Skin Irritation Assay

Skin irritation potential was evaluated by SGS Taiwan Ltd. (Report No. PUG24A00099) using reconstructed human epidermis (EpiSkin, batch 24 EKIN 047) according to OECD Test Guideline 439. Purified cEVs (1 × 10^10^ particles/mL) were topically applied to tissue surfaces for 15 min, followed by thorough rinsing and 42‐h incubation. Tissue viability was assessed by MTT assay; the absorbance of the MTT formazan product at 570 nm was measured using a microplate reader, with 5% SDS and PBS serving as positive and negative controls, respectively. Viability was expressed as percentage relative to negative control, with mean viability > 50% classified as non‐irritating according to UN GHS criteria.

### 2.9 Measurement of Intracellular Melanin Content

To measure intracellular melanin content, MNT‐1 cells were seeded in 6‐well cell culture plates at a density of 5 × 10^5^ cells/well, with each treatment performed in triplicate. After overnight incubation, cells were treated with medium‐diluted cEVs for 72 h. Cells were then carefully harvested using a sterile cell scraper and washed with 500 μL of 1 × PBS. The cell suspension was centrifuged at 3000 × g for 5 min at 4°C, and the PBS supernatant was removed. Cell pellets were lysed with 200 μL of melanin lysis buffer (1 N NaOH containing 10% DMSO) and heated at 80°C for 90 min to ensure complete solubilization of the highly insoluble melanin granules. The absorbance of the solubilized melanin at 405 nm was measured using a Multiskan GO microplate spectrophotometer (Thermo Fisher Scientific). To account for potential variations in cell density and protein content across treatment groups, melanin absorbance values were normalized to two independent reference measures: total cellular protein content, quantified using the BCA assay (T‐Pro Biotechnology, cat. No. JB04‐D001), and cell number, determined by hemocytometer count prior to harvesting. Relative melanin content was expressed as a percentage of the negative control for each normalization method.

### 2.10 EV Uptake Assay

Purified cEVs were precipitated using Total Exosome Isolation Reagent (T‐Pro Biotechnology, cat. No. JO66‐V002S). The precipitated pellet was resuspended in DiO staining working solution (Servicebio, cat. No. G1704) and incubated for 30 min at 37°C. Labeled cEVs were re‐precipitated to remove excess staining solution and resuspended in 1× PBS. For the uptake assay, 3 × 10^5^ HaCaT cells were seeded on coverslips in a 6‐well plate and incubated overnight at 37°C. The following day, 100 μL of DiO‐labeled cEVs was added and cells were incubated for 6 h at 37°C. Coverslips were washed with PBS, fixed with 4% paraformaldehyde for 15 min at room temperature, washed again, and mounted with DAPI‐containing mounting medium. Cellular uptake was visualized using a fluorescence microscope.

### 2.11 Quantitative Real‐Time Polymerase Chain Reaction (qRT‐PCR)

For qRT‐PCR analysis of TYR and TRP‐1 expression, MNT‐1 cells were treated with diluted cEVs as described in intracellular melanin measurement for 72 h. After treatments, cells were harvested and total RNA was extracted using TRIzol reagent (Thermo Fisher Scientific). First strand cDNA was synthesized using SuperScript IV First‐Strand Synthesis System (Thermo Fisher Scientific) and qPCR analysis was performed using SYBR Green Universal Master Mix (Thermo Fisher Scientific). The GAPDH gene was used as the reference gene for the normalization of TYR and TRP‐1 expression. The relative expression levels of target genes were calculated using the 2^(−ΔΔCt) method. Primer sets used for qPCR were listed in Table S1.

### 2.12 Immunoblotting Analysis

Immunoblotting was performed to detect plant extracellular vesicle‐associated marker proteins (PEN1 and TET8) in purified cEVs, and to assess TYR and TRP‐1 protein expression in cEV‐treated MNT‐1 cells. For plant EV marker detection, purified cEVs were lysed in RIPA lysis buffer, and cleared lysates were collected by centrifugation at 12000 × g for 15 min at 4°C. For TYR and TRP‐1 analysis, MNT‐1 cells were treated with diluted cEVs for 72 h, harvested, and lysed in RIPA lysis buffer, and cleared cell lysates were collected by centrifugation. In both cases, protein concentrations were quantified using the BCA assay (T‐Pro Biotechnology, cat. No. JB04‐D001). Lysates were resolved on a 10% acrylamide gel under reducing conditions and transferred to a PVDF membrane (Merck Millipore, Burlington, MA). Membranes were blocked with 5% skim milk and incubated with primary antibodies against PEN1 (CUSABIO, cat. No. CSB‐PA875527XA01DOA), TET8 (CUSABIO, cat. No. CSB‐PA212091XA01DOA), TYR (Genetex, cat. No. GTX04909), or TRP‐1 (Cell Signaling Technology, cat. No. 58193), followed by a horseradish peroxidase‐conjugated goat anti‐rabbit secondary antibody (Genetex, cat. No. GTX213110‐01). Protein bands were visualized using SuperSignal West Pico PLUS chemiluminescent substrate (Thermo Fisher Scientific).

### 2.13 Statistical Analysis

All experiments were performed in triplicate with at least three independent replicates. Data are expressed as mean ± SD. Statistical comparisons were performed using one‐way ANOVA. Differences were considered statistically significant at *p < 0.05, **p < 0.01, ***p < 0.001, and ****p < 0.0001. For the in vitro skin irritation assay (SGS Taiwan Ltd., OECD TG 439), tissue viability > 50% was classified as non‐irritating according to UN GHS criteria. Statistical analyses were conducted using GraphPad Prism version 10.6.1.

## 3 Results

### 3.1 Isolation and Purification of Cucumber‐Derived Exosome‐Like Vesicles

**FIGURE 1:**
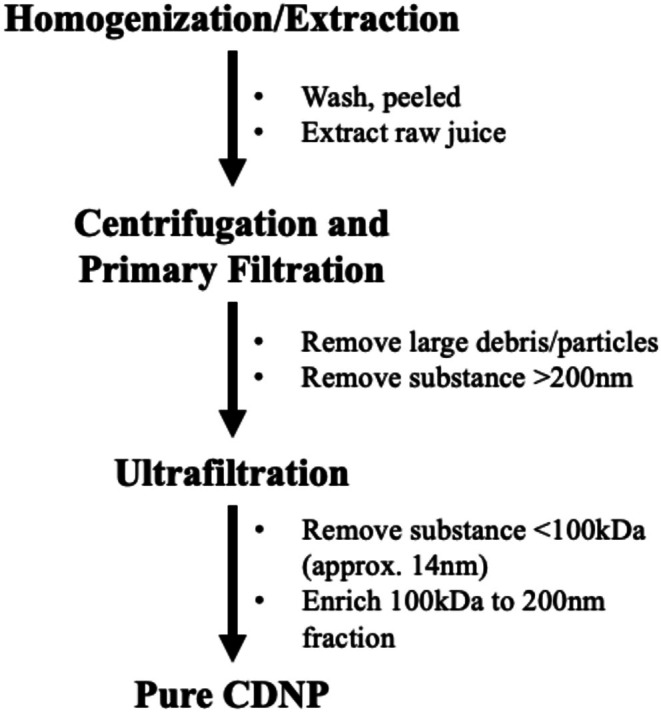
Schematic workflow for the isolation and purification of cEVs. Flowchart illustrating the isolation procedure for cEVs from fresh cucumbers. Following homogenization and extraction of raw juice, centrifugation and filtration (0.45 μm) remove large debris and particles > 200 nm. Ultrafiltration through a 100‐kDa MWCO membrane removes molecules < 100 kDa (~14 nm) and enriches the 100‐kDa to 200‐nm vesicle fraction, yielding purified CDNPs.

To obtain cEVs for subsequent characterization and functional studies, we employed a multi‐step isolation protocol combining differential centrifugation, filtration, and ultrafiltration techniques (Figure 1). Fresh cucumbers were washed and peeled to remove surface contaminants and external tissues. The peeled cucumber flesh was homogenized to extract raw juice for cEV isolation.

The isolation procedure consisted of three sequential steps. First, the raw cucumber juice underwent centrifugation and primary filtration to remove large cellular debris and particles. This step involved low‐speed centrifugation followed by filtration through a 0.45‐μm membrane, effectively eliminating particles larger than 200 nm. Second, the clarified filtrate was subjected to ultrafiltration using a 100‐kDa MWCO membrane. This critical step served dual purposes: removal of small molecules and soluble proteins below 100 kDa (approximately 14 nm in diameter), and enrichment of the 100‐kDa to 200‐nm size fraction, which corresponds to the expected size range of extracellular vesicles, including exosomes. Finally, cEVs retained on the ultrafiltration membrane were recovered through repeated washing with PBS, followed by a final centrifugation step to remove any remaining insoluble aggregates.

This systematic isolation approach yielded purified cucumber‐derived nanoparticles (CDNP), representing a highly enriched population of cEVs suitable for downstream analyses. The combination of size‐based separation methods ensured the removal of contaminating cellular components while preserving the integrity of extracellular vesicles within the target size range characteristic of plant‐derived exosomes.

### 3.2 Characterization of Cucumber‐Derived Exosome‐Like Vesicles by Nanoparticle Tracking Analysis

**FIGURE 2:**
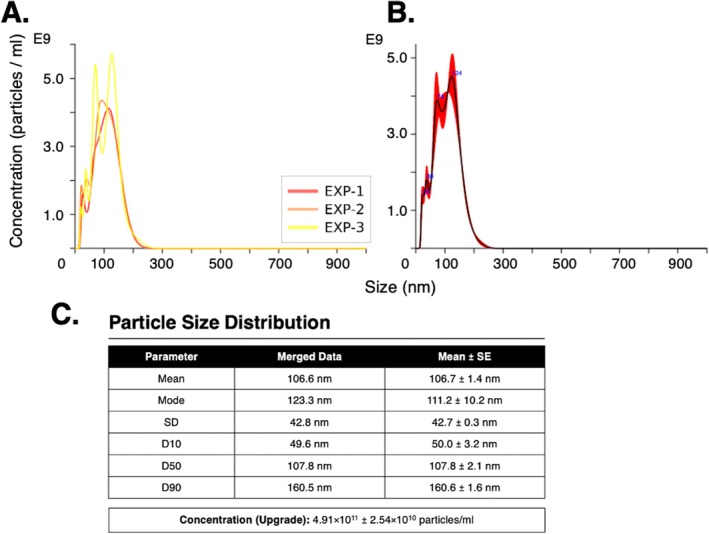
Nanoparticle tracking analysis of cEVs. (A) Size distribution profiles from three independent measurements showing reproducible particle populations (colored lines represent individual technical replicates). (B) Averaged size distribution with error bars representing ±1 standard error of the mean (SEM). (C) The table displays merged data statistics and mean values ± standard error (SE) from three technical replicates. D10, D50, and D90 represent the 10th, 50th (median), and 90th percentile values of the size distribution, respectively.

To characterize the physical properties of the isolated cEVs, NTA was performed in triplicate to determine size distribution and concentration. The merged data revealed a relatively homogeneous population of nanoparticles with characteristics consistent with extracellular vesicles (Figure 2A,B).

The size distribution analysis demonstrated that cEVs exhibited a characteristic profile consistent with extracellular vesicles (Figure 2C). The mean diameter of cEVs was 106.7 ± 1.4 nm. The standard deviation (SD) of the size distribution was 42.7 ± 0.3 nm, indicating moderate size heterogeneity within the population. Further statistical analysis of the size distribution revealed that D10, D50, and D90 values (representing the diameters at which 10%, 50%, and 90% of the particles are smaller, respectively) were 50.0 ± 3.2 nm, 107.8 ± 2.1 nm, and 160.6 ± 1.6 nm, respectively. These values confirmed that the majority of cEVs fell within the 50–160 nm size range, characteristic of exosome‐like vesicles. The concentration was determined to be 4.91 × 10^11^ ± 2.54 × 10^10^ particles/mL, demonstrating successful vesicle enrichment.

These NTA results confirmed successful isolation of cEVs with size characteristics consistent with plant‐derived exosomes. The relatively narrow size distribution, high reproducibility across three independent measurements, and high particle concentration indicated that the purification protocol effectively enriched extracellular vesicles while minimizing contamination from larger cellular debris or protein aggregates.

### 3.3 Morphological, Protein Profile, and EV Marker Characterization of Cucumber‐Derived Exosome‐Like Vesicles

**FIGURE 3:**
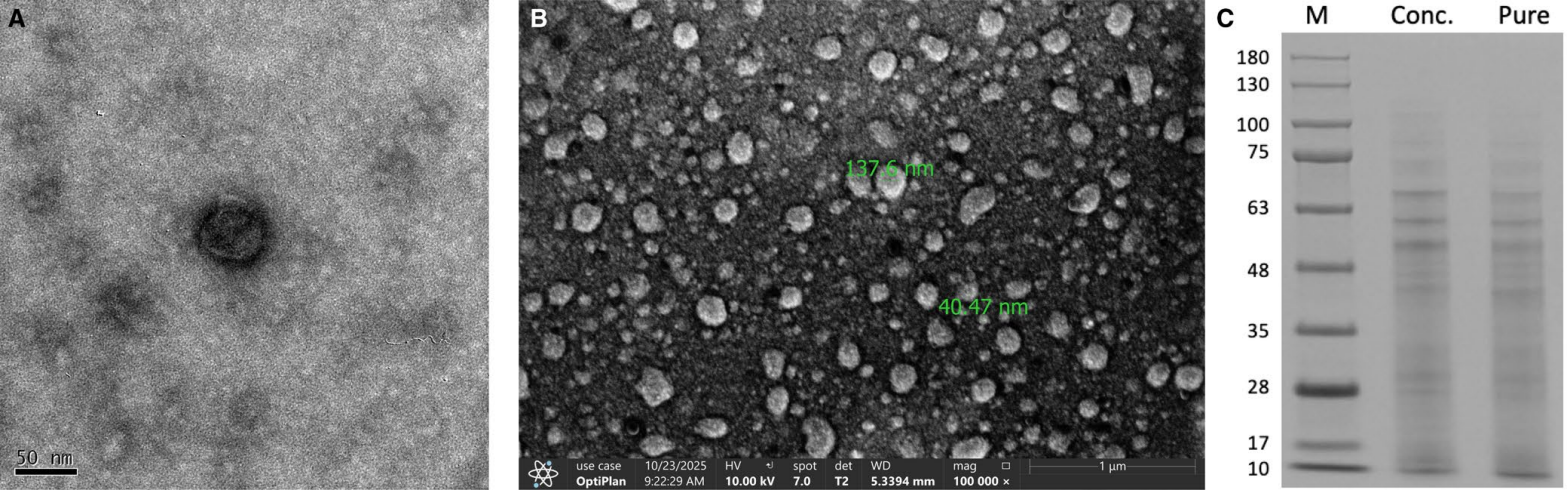
Morphological and protein profile characterization of cEVs. (A) TEM image of purified cEVs. Representative TEM micrograph showing a vesicle with an intact lipid bilayer membrane. Scale bar: 50 nm. (B) Representative SEM micrograph showing the distribution and morphology of cEVs at 100 kx magnification. Scale bar: 1 μm. (C) Protein profiles of concentrated cucumber lysate (Conc.) and purified cEVs (Pure) visualized by SDS‐PAGE with Coomassie blue staining. The M lane shows the protein molecular weight marker.

To further characterize the isolated cEVs and evaluate purification effectiveness, TEM, SEM, and SDS‐PAGE analyses were performed (Figure 3). TEM analysis revealed membrane‐bound vesicular structures with morphological characteristics consistent with exosome‐like vesicles (Figure 3A). The cEVs displayed spherical morphology typical of extracellular vesicles under negative staining, with well‐defined lipid bilayer membranes appearing as darker‐stained peripheral regions surrounding lighter interiors. SEM analysis showed individual vesicles displaying spherical morphology with representative size measurements (137.6 and 40.47 nm) consistent with the exosome size range (Figure 3B). Individual vesicles ranged from approximately 50 to 150 nm in diameter, consistent with NTA measurements.

SDS‐PAGE analysis compared concentrated cucumber lysate with purified cEVs (Figure 3C). The concentrated lysate displayed a complex protein profile with multiple prominent bands across a broad molecular weight range, indicating abundant cytosolic and structural proteins. In contrast, purified cEVs exhibited a simplified profile with fewer visible bands, demonstrating effective removal of contaminating cellular proteins during purification. The reduced band complexity and intensity indicated successful depletion of highly abundant soluble proteins not associated with extracellular vesicles, while retention of specific bands confirmed the presence of vesicle‐associated proteins.

**FIGURE 4:**
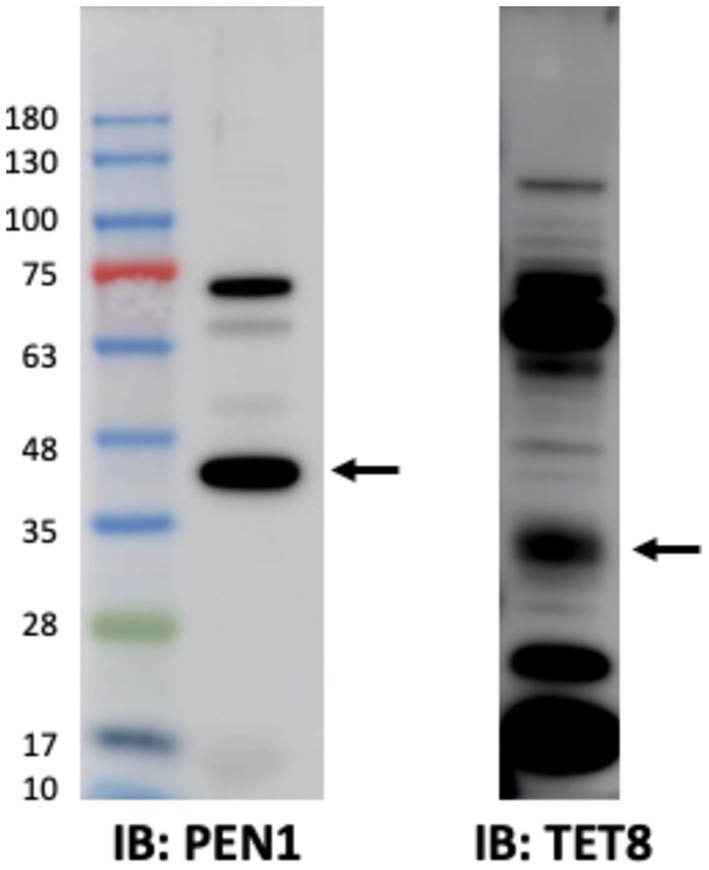
Immunoblotting Confirmation of Plant EV Marker Expression in Purified cEVs. Western blot analysis of purified cEVs probed with antibodies against plant extracellular vesicle marker proteins PEN1 (left panel) and TET8 (right panel). Arrows indicate bands at the expected molecular weights for PEN1 (~45 kDa) and TET8 (~37 kDa).

These results confirmed successful isolation of cEVs with intact membrane structures and enriched vesicular protein content. The combined TEM, SEM, and SDS‐PAGE analyses demonstrated that the purification protocol effectively yielded a vesicle‐enriched fraction suitable for subsequent functional analyses. To further validate the vesicular identity of purified cEVs and address concerns regarding co‐isolation of non‐vesicular plant nanoparticles, immunoblotting was performed using antibodies against two established plant EV marker proteins: PEN1 and TET8. Western blot analysis confirmed the presence of both PEN1 and TET8 in purified cEVs, with bands detected at their respective expected molecular weights (Figure 4). The detection of these plant‐specific EV‐associated proteins provides independent biochemical evidence that the isolated nanoparticles are bona fide extracellular vesicles rather than non‐vesicular co‐isolated plant nanoparticles, thereby supporting the vesicular purity of the cEV preparation.

### 3.4 Cytotoxicity and Skin Irritation Assessment of Cucumber‐Derived Exosome‐Like Vesicles

**FIGURE 5:**
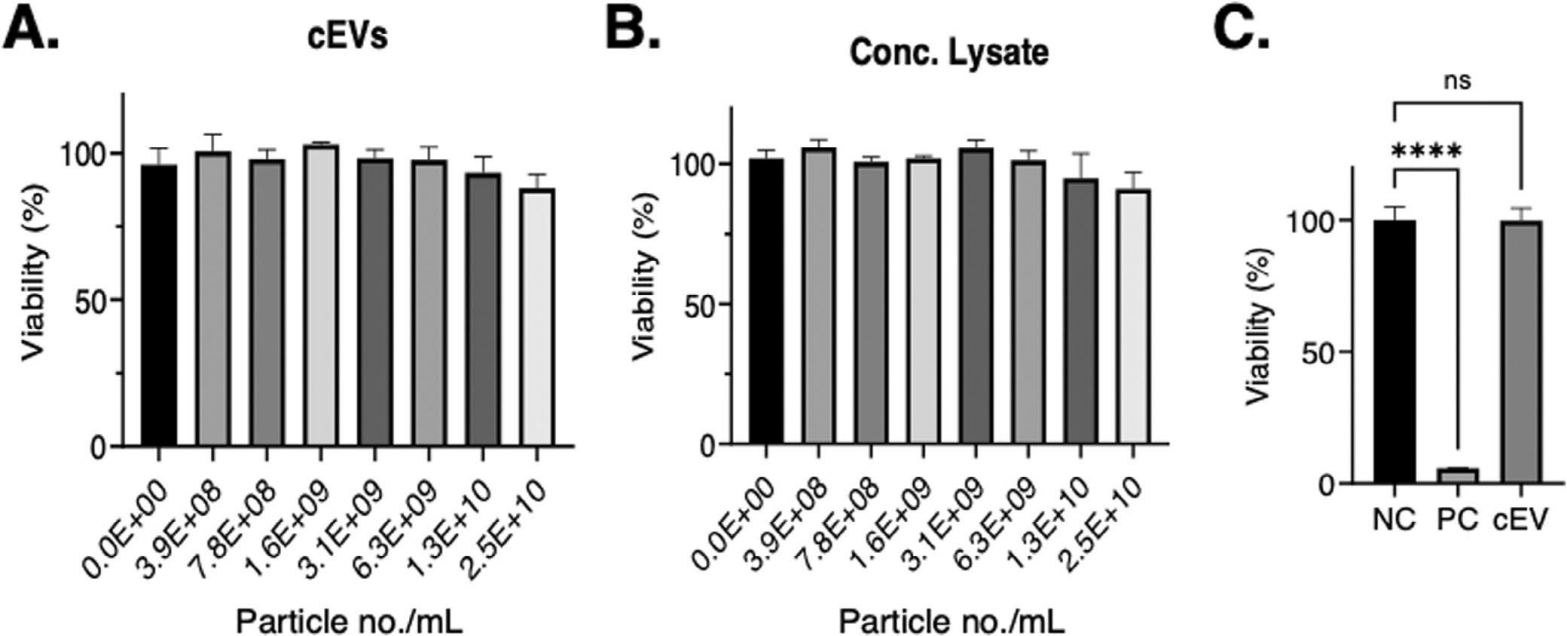
Biosafety assessment of cucumber‐derived exosome‐like vesicles. MNT‐1 cell viability following 72‐h treatment with (A) purified cEVs or (B) concentrated cucumber lysate (3.9 × 10^8^ to 2.5 × 10^10^ particles/mL), measured by MTS assay (formazan absorbance at 490 nm). (C) Reconstructed human epidermis tissue viability following 15‐min cEV exposure (1 × 10^10^ particles/mL) and 42‐h incubation, measured by MTT assay (formazan absorbance at 570 nm). PC: 5% SDS positive control; NC: PBS negative control. Data are presented as mean ± SEM from three independent experiments (*n* = 3).

To evaluate the biosafety of cEVs for therapeutic applications, cytotoxic effects of purified cEVs and concentrated cucumber lysate were assessed on MNT‐1 cells using the MTS assay. Cells were treated with various concentrations (3.9 × 10^8^ to 2.5 × 10^10^ particles/mL) for 72 h before viability measurement (Figure 5A,B).

Both purified cEVs and concentrated lysate induced no statistically significant reduction in cell viability across the tested concentration range. Cell viability remained consistently above 90% for purified cEVs and concentrated lysate at all concentrations relative to untreated controls. The comparable cytotoxicity profiles between purified cEVs and concentrated lysate suggested that the absence of cytotoxic effects is a consistent property of cucumber‐derived preparations maintained throughout purification.

To further assess skin safety, in vitro skin irritation testing was conducted using reconstructed human epidermis according to OECD testing guideline. Following 15‐min topical application of cEVs (1 × 10^10^ particles/mL) and 42‐h incubation, tissue viability was assessed by measuring the absorbance of the MTT formazan product at 570 nm. The results demonstrated 99.9% tissue viability (mean ± SD: 99.9% ± 4.7%), compared to 100.0% ± 5.0% for the negative control (PBS) and 5.7% ± 0.0% for the positive control (5% SDS) (Figure 5C). Based on tissue viability exceeding 50%, cEVs were classified as non‐irritating (UN GHS No Category), indicating no skin irritation potential.

These results demonstrated that cEVs induced no statistically significant reduction in MNT‐1 cell viability across concentrations spanning more than two orders of magnitude and were classified as non‐irritating to reconstructed human skin tissue per UN GHS criteria, supporting their in vitro safety within the tested concentration range for further preclinical evaluation.

### 3.5 Cellular Uptake of cEVs by HaCaT Keratinocytes

**FIGURE 6:**
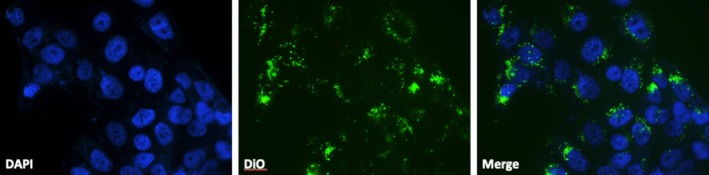
Cellular uptake of DiO‐labeled cEVs by HaCaT human keratinocytes. Representative fluorescence microscopy images showing the uptake of cEVs by HaCaT cells. Purified cEVs were labeled with the lipophilic fluorescent dye DiO (green) and incubated with HaCaT cells for 6 h at 37°C. Nuclei were counterstained with DAPI (blue).

To investigate whether cEVs could be internalized by human keratinocytes, we performed a fluorescence microscopy‐based uptake assay using DiO‐labeled cEVs and HaCaT cells, an immortalized human keratinocyte line widely used as a surrogate model for the epidermal barrier. Following 6 h incubation with DiO‐labeled cEVs, fluorescence microscopy revealed distinct punctate green fluorescent signals within the cytoplasm, with prominent accumulation in the perinuclear region, while DAPI counterstaining confirmed nuclear integrity (Figure 6). These findings confirm that cEVs are efficiently internalized by human keratinocytes, providing direct experimental evidence for their endocytic internalization in a monolayer culture model.

### 3.6 Depigmentation Effects of Cucumber‐Derived Exosome‐Like Vesicles on MNT‐1 Melanoma Cells

**FIGURE 7:**
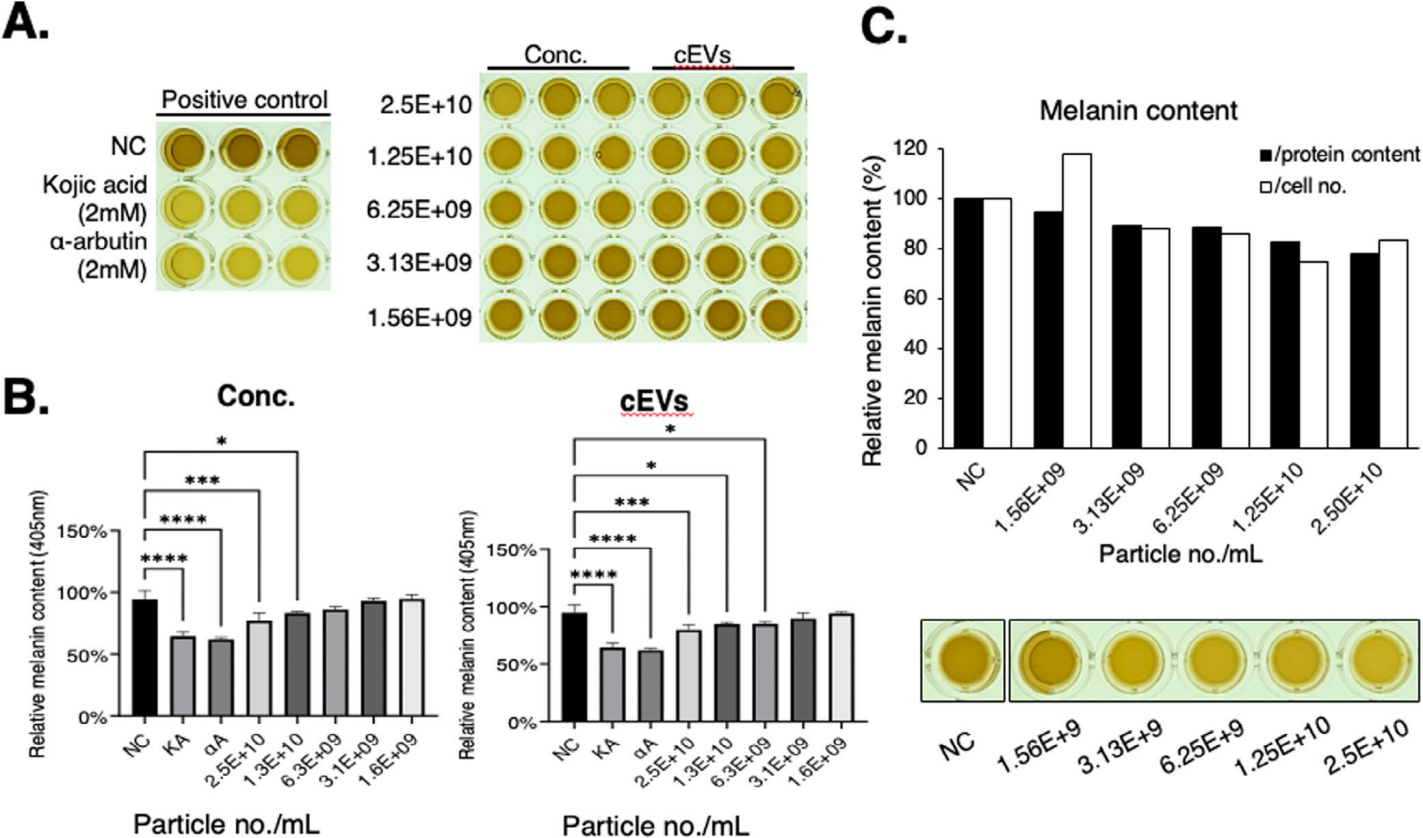
Melanin‐inhibitory effects of cEVs and concentrated lysate in MNT‐1 melanoma cells. Depigmentation activity of concentrated cucumber lysate and purified cEVs in MNT‐1 melanoma cells following 72‐h treatment. (A) Visual assessment of melanin production in harvested cell pellets of positive controls (kojic acid 2 mM and α‐arbutin 2 mM), concentrated lysate (Conc.), and purified cEVs at indicated particle concentrations. (B) Quantification of relative melanin content following treatment with concentrated lysate (left) or purified cEVs (right) at indicated particle concentrations. Kojic acid (KA) and α‐arbutin (αA) served as positive controls; untreated cells served as NC. Melanin absorbance was quantified at 405 nm and normalized to NC (set at 100%). Data are presented as mean ± SD from three independent experiments (*n* = 3). Statistical significance: *p < 0.05, ***p < 0.001, ****p < 0.0001 compared to NC. (C) Normalization of intracellular melanin content in cEV‐treated MNT‐1 cells to total protein content (black bars) or cell number (white bars), expressed as percentage of NC. Visual assessment of corresponding cell pellets is shown below.

To investigate the depigmentation effects of cEVs, MNT‐1 cells were treated with various concentrations of concentrated cucumber lysate or purified cEVs for 72 h, and intracellular melanin content was quantified. Kojic acid (2 mM) and α‐arbutin (2 mM) served as positive controls, while untreated cells served as the negative control (NC) (Figure 7). Visual inspection revealed concentration‐dependent depigmentation in both lysate‐ and cEV‐treated groups (Figure 7A). To ensure that the observed melanin reduction reflects genuine depigmentation rather than differences in cell density or protein content, melanin absorbance values were normalized to both total protein content and cell number. Both normalization strategies yielded consistent, concentration‐dependent reductions in intracellular melanin content following cEV treatment, with the highest concentration (2.5 × 10^10^ particles/mL) reducing normalized melanin to approximately 75% of the negative control under both reference conditions (Figure 7C). Cells treated with kojic acid and α‐arbutin exhibited noticeably lighter pigmentation compared to NC, confirming positive control efficacy. Both concentrated lysate and purified cEVs induced visible melanin reduction that became more apparent at higher concentrations.

Quantitative analysis showed that kojic acid and α‐arbutin significantly reduced melanin levels to approximately 60%–65% of NC (****p < 0.0001), while both cucumber preparations exhibited dose‐dependent melanin reduction (Figure 7B). At the highest concentration (2.5 × 10^10^ particles/mL), both lysate and cEVs decreased melanin to approximately 75% of NC (***p < 0.001). At 1.3 × 10^10^ particles/mL, both showed significant reduction (*p < 0.05). Notably, at 6.3 × 10^9^ particles/mL, only purified cEVs exhibited significant melanin reduction (*p < 0.05), suggesting enhanced potency at sub‐optimal concentrations. At lower concentrations (3.1 × 10^9^ and 1.6 × 10^9^ particles/mL), both preparations showed melanin levels comparable to NC, indicating a concentration threshold.

These results demonstrated that both cucumber preparations possess statistically significant, concentration‐dependent depigmentation activity, with purified cEVs exhibiting greater potency at intermediate concentrations. The comparable efficacy at higher concentrations and absence of statistically significant cytotoxicity (Figure 5) support their potential as preliminary in vitro depigmentation candidates with an acceptable in vitro safety profile.

### 3.7 Effects of Cucumber‐Derived Exosome‐Like Vesicles on Melanogenic Gene and Protein Expression

To elucidate the molecular mechanism underlying the melanin‐inhibitory effects observed in Figure 7, we examined mRNA and protein expression of TYR and TRP‐1 following cEV treatment. TYR is the rate‐limiting enzyme in melanin biosynthesis, while TRP‐1 plays a critical role in eumelanin processing and tyrosinase stabilization.

**FIGURE 8:**
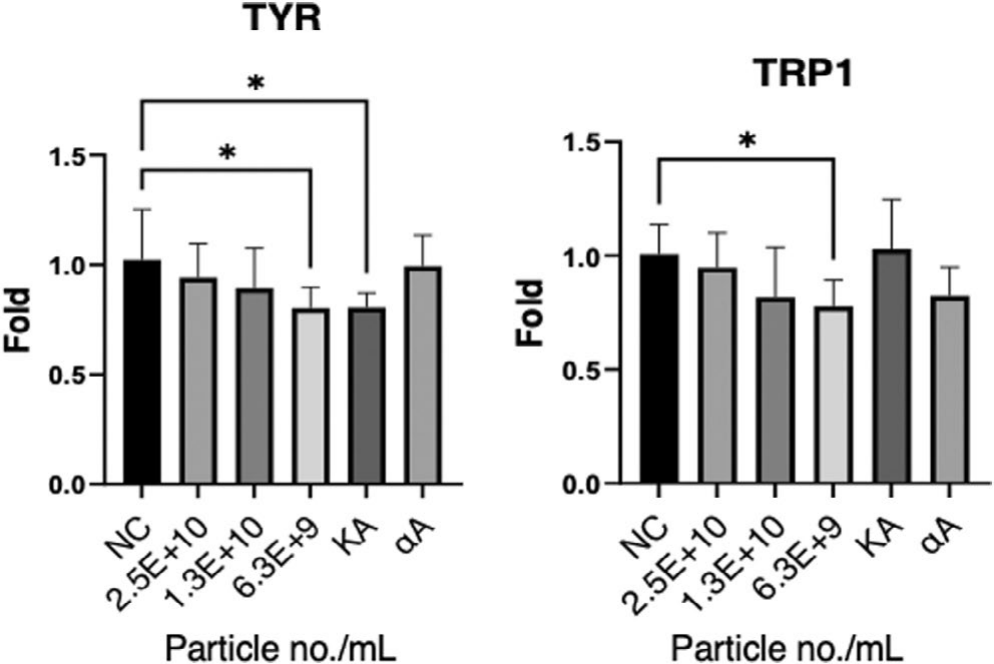
cEVs downregulate melanogenesis‐related genes TYR and TRP‐1. qRT‐PCR analysis of (left panel) TYR and (right panel) TRP‐1 mRNA expression in MNT‐1 melanoma cells following 72‐h treatment with purified cEVs at indicated concentrations. Gene expression levels were normalized to GAPDH housekeeping gene and expressed as fold change relative to NC (untreated cells). Data are presented as mean ± SD from three independent experiments (*n* = 3). Asterisks (*) indicate statistically significant differences compared to NC (*p < 0.05).

**FIGURE 9:**
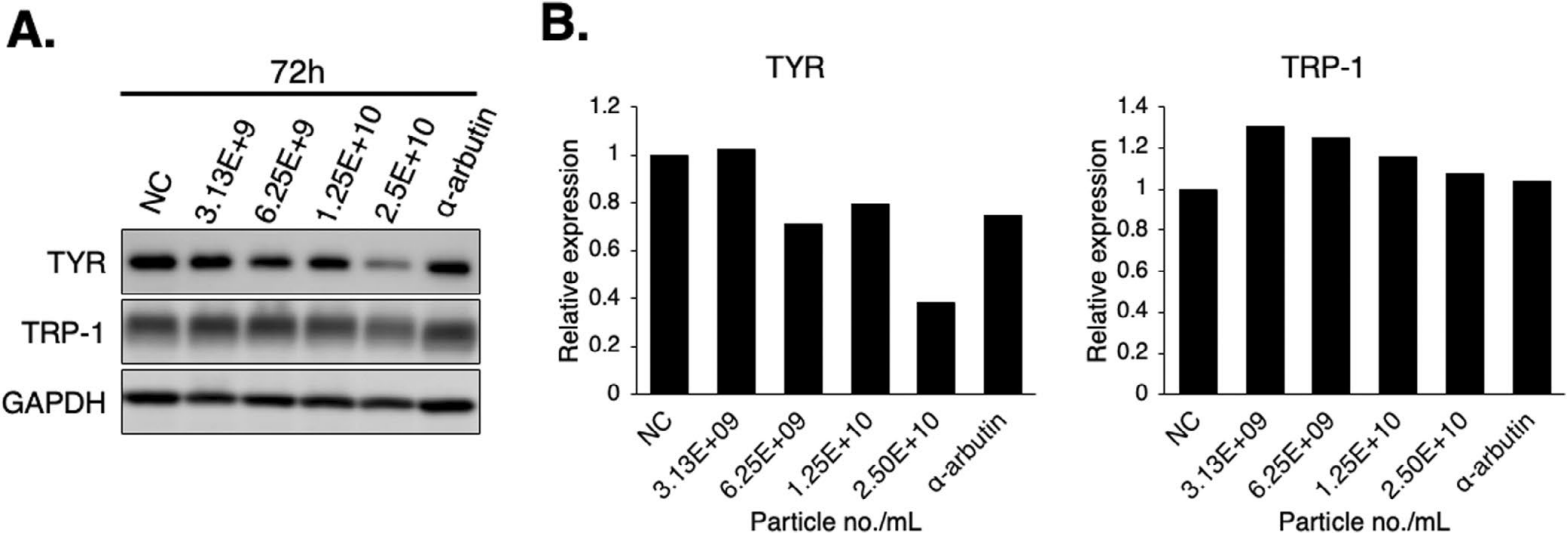
cEV treatment reduces TYR protein expression in MNT‐1 cells in a dose‐dependent manner. Immunoblotting analysis of TYR and TRP‐1 protein expression in MNT‐1 melanoma cells following 72‐h treatment with purified cEVs at the indicated concentrations, with α‐arbutin as a positive control. (A) Representative Western blot images showing TYR, TRP‐1, and GAPDH (loading control) protein bands. (B) Densitometric quantification of TYR (left) and TRP‐1 (right) protein expression normalized to GAPDH, expressed relative to the negative control (NC). Densitometric data represent a single representative experiment and are therefore presented as preliminary.

**FIGURE 10:**
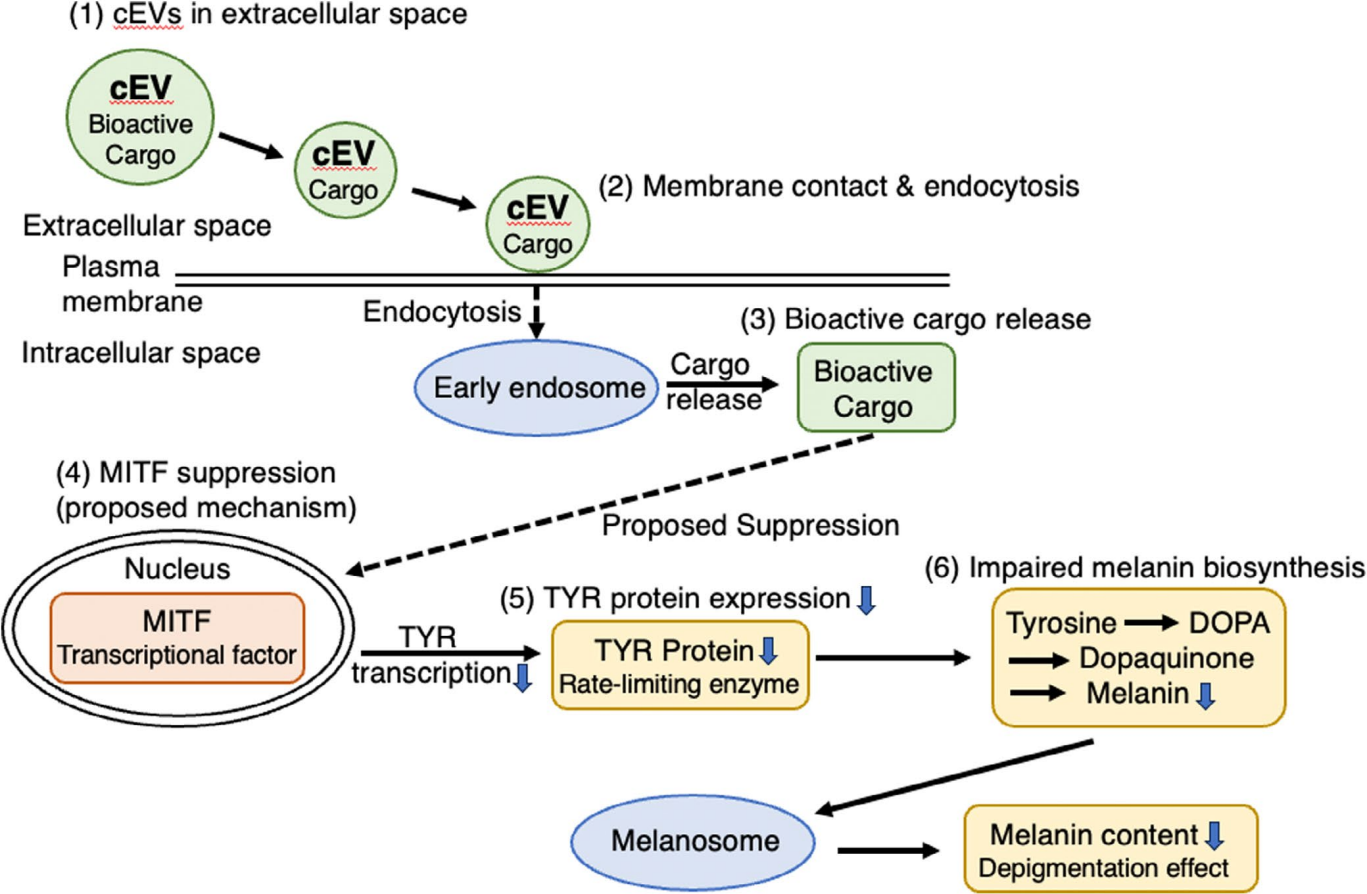
Proposed mechanism of cEV‐mediated melanogenesis inhibition. Schematic illustration of the proposed mechanisms by which cEVs inhibit melanogenesis in melanocytes. (1) cEVs in the extracellular environment approach and contact the melanocyte plasma membrane. (2) cEVs are internalized via endocytosis into early endosomes. (3) Bioactive cargo is released intracellularly. (4) Released cargo is proposed to suppress MITF transcriptional activity (dashed line; proposed mechanism not directly demonstrated). (5) Reduced MITF activity decreases TYR transcription, leading to dose‐dependent TYR protein downregulation as demonstrated by immunoblotting; TRP‐1 protein expression is relatively preserved. (6) Reduced TYR enzymatic activity impairs conversion of tyrosine to DOPA and downstream melanin biosynthesis, resulting in reduced melanosome melanin content and net depigmentation.

qRT‐PCR analysis revealed modest reductions in TYR and TRP‐1 mRNA expression following cEV treatment (Figure 8). For TYR, a statistically significant reduction was observed at 6.3 × 10^9^ particles/mL (*p < 0.05 vs. NC), with expression reduced to approximately 80% of NC. Similarly, TRP‐1 mRNA showed a statistically significant reduction at 6.3 × 10^9^ particles/mL (*p < 0.05 vs. NC), with expression reduced to approximately 75% of NC. To further elucidate the molecular basis of cEV‐mediated depigmentation at the protein level, immunoblotting for TYR and TRP‐1 was performed following 72‐h cEV treatment (Figure 9). In a representative experiment, immunoblotting revealed a trend toward dose‐dependent reduction in TYR protein expression, reaching approximately 40% of NC at the highest concentration tested (2.5 × 10^10^ particles/mL)—a more pronounced reduction than observed at the mRNA level; however, as these data are derived from a single representative experiment, this observation should be considered preliminary and the post‐transcriptional interpretation remains speculative, pending confirmation with full biological replicates. In contrast, TRP‐1 protein levels remained relatively stable across all concentrations, indicating that cEV‐mediated effects are preferentially directed toward TYR regulation. A schematic illustration summarizing the proposed mechanisms of cEV‐mediated melanogenesis inhibition is presented in Figure 10.

## 4 Discussion

This study represents the first investigation into the isolation, characterization, and melanin‐inhibitory effects of cEVs. We successfully isolated cEVs from fresh cucumber juice using a systematic protocol combining differential centrifugation, filtration, and ultrafiltration. The purified cEVs exhibited characteristics of plant‐derived exosomes. NTA revealed a homogeneous size distribution centered around 100–110 nm, with D10, D50, and D90 values confirming that the majority of vesicles fell within the 50–200 nm exosome size range with minimal contamination from larger debris. TEM and SEM validated intact membrane‐enclosed vesicles, confirming preservation of vesicular integrity. SDS‐PAGE showed substantial differences between concentrated lysate and purified cEVs, with the purified fraction displaying reduced complexity and depletion of abundant soluble proteins. Furthermore, immunoblotting detection of the plant EV marker proteins PEN1 and TET8 in purified cEVs provides independent biochemical evidence that the isolated nanoparticles are bona fide extracellular vesicles rather than non‐vesicular co‐isolated plant nanoparticles, thereby supporting the vesicular purity of the cEV preparation.

It should be noted that the term “exosome‐like vesicles” is used throughout this manuscript to describe nanoparticles sharing size, morphological, and biochemical characteristics with exosomes, rather than to imply confirmed endosomal biogenesis [16]. Definitive classification as true exosomes would require direct evidence of multivesicular body‐mediated biogenesis, which was beyond the scope of the present study. The use of plant‐specific EV marker proteins PEN1 and TET8 provides biochemical support for the extracellular vesicle identity of the isolated preparations, consistent with MISEV2018 recommendations to demonstrate the presence of EV‐associated marker proteins as part of rigorous EV characterization [16].

Notably, cEVs demonstrated statistically significant, concentration‐dependent melanin inhibition in MNT‐1 cells, achieving efficacy comparable to kojic acid and α‐arbutin without inducing statistically significant cytotoxicity within the tested concentration range. These results provide preliminary proof‐of‐concept evidence supporting the depigmentation potential and biosafety of cEVs, warranting further investigation in more physiologically relevant model systems.

A critical consideration for any cosmetic or therapeutic agent is biosafety. Our cytotoxicity assessment revealed that both purified cEVs and concentrated cucumber lysate induced no statistically significant reduction in MNT‐1 cell viability across concentrations spanning more than two orders of magnitude (3.9 × 10^8^ to 2.5 × 10^10^ particles/mL), with cell viability consistently above 90% and no statistically significant cytotoxic effects.

This absence of detectable cytotoxicity within the tested concentration range contrasts markedly with the documented toxicity profiles of conventional skin‐lightening products. Mercury‐containing cosmetics can contain levels exceeding 19 000 ppm (ppm), far above the FDA safety limit of 1 ppm, leading to neurologic damage, contact dermatitis, and mercury poisoning [17]. A systematic review found that approximately 25% of 787 analyzed products exceeded the 1 μg/g regulatory limit, with concentrations reaching 314 387 μg/g, and biomarker analyses revealed that 65%–70% of 863 users exceeded reference values for mercury in urine, blood, and hair [18]. Additionally, unsupervised hydroquinone use can cause ochronosis, contact dermatitis, and systemic complications [19].

The absence of cytotoxicity for cEVs, combined with their derivation from an edible plant with established dietary consumption history, suggests a more favorable in vitro cytotoxicity profile relative to conventional agents within the tested parameters. The comparable cytotoxicity profiles between purified cEVs and concentrated lysate suggest that the absence of cytotoxic effects is a consistent property of cucumber‐derived components maintained throughout purification.

A principal finding of this study is the statistically significant, concentration‐dependent melanin‐inhibitory activity of cEVs. Quantitative analysis demonstrated that both concentrated cucumber lysate and purified cEVs produced dose‐dependent depigmentation, with the highest concentration (2.5 × 10^10^ particles/mL) reducing melanin to approximately 75% of control, comparable to kojic acid and α‐arbutin. Notably, at 6.3 × 10^9^ particles/mL, only purified cEVs exhibited statistically significant melanin reduction while the concentrated lysate did not, suggesting that the purification and enrichment of bioactive components within the vesicular fraction confers enhanced potency relative to crude cucumber extract. This finding supports the added value of the vesicle isolation process and highlights that the depigmentation activity observed is not simply attributable to soluble components present in unprocessed cucumber extract. The concentration‐dependent response and comparable efficacy at higher doses indicate that melanin‐inhibitory factors are present throughout cucumber extract, with enrichment in the vesicular fraction.

The depigmentation activity of cEVs can be contextualized within the broader landscape of naturally derived skin‐lightening agents. Conventional depigmenting agents, including hydroquinone, kojic acid, and arbutin, are associated with adverse effects such as erythema, skin peeling, and dryness, driving interest in naturally derived alternatives [20]. Among these, white birch sap (WBS) has demonstrated dose‐dependent TYR inhibition and melanin reduction in B16F10 cells and zebrafish larvae with minimal skin irritation [21], while natural phenolic compounds—including flavonoids, hydroxystilbenes, and oligomeric procyanidins—exhibit TYR inhibitory activity through competitive inhibition, antioxidant mechanisms, and MITF pathway modulation [20]. The melanin‐inhibitory activity and TYR protein downregulation observed in the present study are broadly comparable to efficacy reported for these agents in the present in vitro model system, with a similarly acceptable in vitro safety profile within the tested conditions. Notably, cEVs deliver bioactive cargo in a membrane‐enclosed nanoparticle form, which may confer advantages in cellular uptake efficiency and protection of cargo from oxidative degradation relative to free molecules. Nevertheless, direct comparisons under standardized experimental conditions will be necessary to establish the relative efficacy of cEVs versus other naturally derived depigmentation candidates.

To elucidate the molecular mechanisms of cEV‐mediated depigmentation, we examined both mRNA and protein expression of TYR and TRP‐1. While transcriptional changes were modest by qRT‐PCR, immunoblotting provided preliminary protein‐level evidence suggesting dose‐dependent TYR downregulation, with TYR protein reduced to approximately 40% of control at the highest cEV concentration in a representative experiment—a suppression magnitude comparable to the α‐arbutin positive control. This discordance between modest mRNA changes and more pronounced protein reduction may suggest that cEVs act through post‐transcriptional or post‐translational mechanisms, including modulation of protein stability, altered translational efficiency, or enhanced proteasomal degradation of TYR; however, this interpretation remains speculative and requires confirmation with independent biological replicates. The selective suppression of TYR protein with relative preservation of TRP‐1 levels further implies that cEV bioactive cargo may specifically target the rate‐limiting step in melanogenesis rather than broadly disrupting the melanogenic program. Possible upstream mediators include interference with the microphthalmia‐associated transcription factor (MITF) pathway, which governs transcriptional regulation of TYR, as well as modulation of melanosome biogenesis or maturation processes [22, 23]. Future investigations employing direct tyrosinase activity assays, MITF pathway analysis, and comprehensive proteomic profiling will be important to fully delineate the mechanistic basis of cEV‐mediated depigmentation. These proposed mechanistic relationships are summarized schematically in Figure 10.

Beyond depigmentation, cEVs hold broader potential for skincare applications. Cucumber has been traditionally valued in cosmetics for multiple beneficial properties: high water content provides hydration and strengthens the moisture barrier while delivering cooling sensations beneficial for soothing sunburns and irritation; minerals including silica, potassium, and magnesium contribute to cooling and potential anti‐aging effects; and bioactive compounds inhibit melanin synthesis, consistent with our findings [24].

The encapsulation of these components within exosome‐like vesicles offers additional advantages for cosmetic applications. Plant‐derived vesicles may combine the cellular uptake properties of nanoscale carriers with natural origin and an acceptable in vitro safety profile. The intrinsic lipid bilayer composition of cEVs may facilitate efficient cellular internalization and intracellular cargo delivery. Furthermore, the capacity to co‐deliver multiple bioactive compounds could enable synergistic effects, enhancing both depigmentation and additional benefits such as hydration, anti‐inflammation, and antioxidant protection.

To further characterize the cellular interactions of cEVs, we performed a fluorescence‐based uptake assay confirming efficient internalization of DiO‐labeled cEVs by HaCaT human keratinocytes within 6 h (Figure 6). This perinuclear distribution pattern is characteristic of endocytic uptake and endo‐lysosomal trafficking, consistent with previous reports of plant‐derived exosome‐like vesicle internalization in mammalian cells. The capacity of cEVs to cross the keratinocyte plasma membrane is consistent with their lipid bilayer composition, nanoscale dimensions (~107 nm), and fusogenic properties commonly attributed to plant‐derived vesicles, supporting the biological plausibility of intracellular cargo delivery in vitro. It should be noted, however, that cellular internalization in a monolayer culture model does not serve as evidence of transcutaneous permeation across the intact skin barrier. Formal assessment using Franz diffusion cells, ex vivo skin explants, or validated 3D reconstructed skin models was beyond the scope of the present study and remains an important priority for future investigation.

Several limitations deserve discussion. First, while we demonstrated melanin inhibition in MNT‐1 cells, validation in normal human melanocytes and three‐dimensional skin models will be important to confirm generalizability. Second, the specific bioactive components responsible for depigmentation remain to be identified through comprehensive analyses. Third, while qRT‐PCR analysis did not reveal statistically significant changes in TYR and TRP‐1 mRNA expression, protein‐level evidence of TYR downregulation has been demonstrated by immunoblotting; further mechanistic studies including direct tyrosinase activity assays and upstream signaling pathway analyses remain important future directions. Fourth, the physicochemical stability of cEVs under varying environmental conditions, including different pH levels, temperatures, and light exposure, was not evaluated in the present study. Assessment of vesicle stability under these conditions is an important prerequisite for practical cosmetic formulation development, as plant‐derived extracellular vesicles may be susceptible to aggregation, membrane disruption, or cargo degradation under suboptimal storage or application conditions. In the absence of such data, any inference regarding formulation feasibility or commercial applicability of cEVs remains premature. Systematic stability characterization across relevant environmental parameters, alongside compatibility testing within cosmetic formulation matrices, therefore represents a critical and prioritized direction for future investigation prior to any translational or commercial development of cEV‐based depigmentation products. Finally, in vivo efficacy studies are necessary to validate depigmentation effects and assess practical utility for topical applications.

From a regulatory and translational perspective, several considerations warrant discussion for the future development of cEV‐based depigmentation products. In the United States, topically applied products intended to affect skin pigmentation may be regulated as cosmetics under the Federal Food, Drug, and Cosmetic Act (FD&C Act). In the European Union, such products would most likely fall under Regulation (EC) No 1223/2009, requiring a Cosmetic Product Safety Report (CPSR) and notification through the Cosmetic Products Notification Portal (CPNP). Given the nanoscale dimensions of cEVs, additional nanomaterial notification and safety assessment obligations under the EU Cosmetics Regulation may apply, and the evolving guidance frameworks from the International Cooperation on Cosmetics Regulation (ICCR) and ISO will need to be incorporated into any regulatory strategy. Beyond classification, comprehensive safety dossiers will be required, encompassing repeated‐dose dermal toxicity, sensitization assessment (e.g., OECD TG 442C/D/E), phototoxicity evaluation, and genotoxicity testing per Scientific Committee on Consumer Safety (SCCS) Notes of Guidance. Proactive engagement with these regulatory frameworks will be essential to translate the depigmentation efficacy and safety profile demonstrated here into safe and compliant cEV‐based products.

## Author Contributions

Conceptualization and design: C.‐Y.H., W.‐C.C., H.‐S.C., Y.‐C.L., C.‐H.H., D.L., and Y.‐W.C. Methodology: C.‐Y.H., W.‐C.C., D.L., and Y.‐W.C. Software: C.‐Y.H. and W.‐C.C. Validation: D.L. and Y.‐W.C. Formal analysis: C.‐Y.H. and W.‐C.C. Investigation: C.‐Y.H. and W.‐C.C. Resources: D.L. and Y.‐W.C. Writing – original draft preparation: C.‐Y.H., W.‐C.C., D.L. and Y.‐W.C. Writing – review and editing: D.L. and Y.‐W.C. All authors have read and agreed to the published version of the manuscript.

## Ethics Statement

The authors have nothing to report.

## Consent

The authors have nothing to report.

## Conflicts of Interest

The authors declare no conflicts of interest.

## Supporting information


**Table S1:** Primer sequences for qRT‐PCR analysis.

## Data Availability

The data that support the findings of this study are available from the corresponding author upon reasonable request.
