# Supplementary material for: Cucumber‐Derived Exosome‐Like Vesicles With Melanin‐Inhibitory Activity and an Acceptable in Vitro Safety Profile
Source: J Cosmet Dermatol. 2026 Jun 11;25(6):e70980. doi: 10.1111/jocd.70980 (PMC13260878; doi:10.1111/jocd.70980)
Supplement: Supplementary file 1 — Table S1: Primer sequences for qRT‐PCR analysis. [file JOCD-25-e70980-s001.docx]

**Supplementary Table 1.** **Primer sequences for qRT-PCR analysis**

| Gene | Direction | Sequences (5’-3’) |
| --- | --- | --- |
| TYR | Forward | GCA CAG ATG AGT ACA TGG GAG G |
|  | Reverse | CTG ATG GCT GTT GTA CTC CTC C |
| TRP1 | Forward | TCT CAA TGG CGA GTG GTC TGT G |
|  | Reverse | CCT GTG GTT CAG GAA GAC GTT G |
| GAPDH | Forward | GCC ACA TCG CTC AGA CA |
|  | Reverse | GCC CAA TAC GAC CAA ATC C |
